# Branched mannan and xyloglucan as a dynamic duo in plant cell walls

**DOI:** 10.1016/j.tcsw.2023.100098

**Published:** 2023-01-25

**Authors:** Annika Grieß-Osowski, Cătălin Voiniciuc

**Affiliations:** a Independent Junior Research Group–Designer Glycans, Leibniz Institute of Plant Biochemistry, 06120 Halle (Saale), Germany; b Department of Biological Data Science, Heinrich-Heine-University Düsseldorf, Universitätsstraße 1, 40225 Düsseldorf, Germany; c Horticultural Sciences Department, University of Florida, Gainesville, FL 32611, United States

**Keywords:** Plant cell wall, Hemicelluloses, Galactoglucomannan, Xyloglucan, Arabidopsis, XyG, xyloglucan, GGM, galactoglucomannan, CSLA, CELLULOSE SYNTHASE-LIKE A, CSLC, CELLULOSE SYNTHASE-LIKE C, GT, glycosyltransferase

Plant surfaces are encased in cellulose, hemicelluloses, pectin, glycoproteins and additional classes of molecules such as lignin and cuticular waxes. While the actual composition of the extracellular matrix varies remarkably between different plant organs and developmental stages, a complex network of cellulose and hemicelluloses is generally responsible for providing strength as well as guiding the growth of plant cells. Cellulose microfibrils are synthesized directly at the plasma membrane, yet their sustained deposition and organization also depend on the intracellular synthesis and delivery of hemicelluloses such as xyloglucan (XyG), mannan and xylan (Zhang et al., 2021). The substitution of these matrix polysaccharides can directly influence their ability to cross-link cellulose microfibrils (Khodayari et al., 2021). (Gluco)mannan backbones are built of β-1,4-mannosyl (Man) units, sometimes interspersed with β-1,4-glucosyl (Glc) units, while XyG backbone is built exclusively of β-1,4-Glc. The biosynthesis of conserved XyG branches has been extensively investigated by several research groups (reviewed by Julian and Zabotina, 2022), but plant β-mannans appeared to be composed of irregularly distributed glycosidic residues until recently (reviewed by Voiniciuc, 2022). Subsequently, Yu et al. (2022) identified a patterned galactoglucomannan (GGM) containing repeating Glc-Man backbone units and regularly spaced β-Gal-α-Gal disaccharide side chains. Oligosaccharide profiling of primary cell wall (PCW) extracts from several eudicot species, including important crops such as tomato, suggests that β-mannans with conserved molecular patterns are widespread. In this update, we summarize newfound relationships between mannan and XyG decorated with galactose (Gal) and discuss their impact on PCW function. The roles of Gal substitutions on mannans and XyG require further investigation but likely facilitate interactions with other cell wall polymers such as cellulose. Notably, the synthesis of these two classes of hemicelluloses relies on a common set of Carbohydrate-Active Enzyme (CAZy; https://www.cazy.org/) families (Table 1).

**Table 1:** Comparison of Arabidopsis GT enzymes for GGM and XyG biosynthesis. The list of characterized CAZy proteins is based on several publications (Julian and Zabotina, 2022, Pauly and Keegstra, 2016, Voiniciuc, 2022, Yu et al., 2022). For the GT34 and GT47 isoforms, the primary enzyme specificity is listed. New abbreviations: Fuc, fucose; GalA, galacturonic acid. Question marks indicate that no related enzymes have been identified for a particular polymer thus far.

	**Xyloglucan (XyG)**		**Galactoglucomannan (GGM)**	
	**Enzyme**	**AGI ID**	**Enzyme**	**AGI ID**
GT2	CSLC4 CSLC5 CSLC6 CSLC8 CSLC12	At3g28180 At4g31590 At3g07330 At2g24630 At4g07960	CSLA2 CSLA3 CSLA7 CSLA9	At5g22740 At1g23480 At2g35650 At5g03760
GT34	XXT1 (α-1,6-Xyl)XXT2 (α-1,6-Xyl)XXT5 (α-1,6-Xyl)	At3g62720 At4g02500 At1g74380	MAGT/MUCI10 (α-1,6-Gal)	At2g22900
GT37	MUR2 / FUT1 (α-1,2-Fuc)	At2g03220	?	?
GT47	MUR3 (β-1,2-Gal)XLT2 (β-1,2-Gal)XUT1 (β-1,2-GalA)	At2g20370 At5g62220 At1g63450	MBGT1 (β-1,2-Gal)	At4g13990
GT106	?	?	MSR1 MSR2	At3g21190 At1g51630

## Mannan and XyG elongation

While β-mannans likely represent the most ancient hemicellulose in the plant kingdom, XyG emerged as the dominant hemicellulose in the PCWs of most angiosperms (Scheller and Ulvskov, 2010). Nevertheless, in some walls such as the softwood of gymnosperms, GGM accumulates as the most abundant hemicellulose after cell expansion is completed (Voiniciuc, 2022). In *Arabidopsis thaliana* (Arabidopsis hereafter), five *CELLULOSE SYNTHASE-LIKE C* (*CSLC*) genes are functionally redundant for the elongation of XyG (Kim et al., 2020), while at least four of the nine *CELLULOSE SYNTHASE-LIKE A* (*CSLA*) genes encode (gluco)mannan synthases (Voiniciuc, 2022). Plant β-1,4-glucan or mannan polymers can be elongated in yeast using CSLC (Cocuron et al., 2007, Robert et al., 2021) or CSLA expression (Robert et al., 2021, Voiniciuc et al., 2019), respectively. One notable difference between these integral Golgi enzymes is that CSLAs use GDP-Man and GDP-Glc as sugar donors, while CSLCs utilize UDP-Glc. Furthermore, their catalytic domains were reported to have opposite orientations (Davis et al., 2010), which would influence nucleotide sugar accessibility and the need for product translocation. Structural prediction of CSLCs identified a “VET” amino acid motif instead of a “TED” sequence, important for product elongation and translocation by cellulose synthases (Julian and Zabotina, 2022). All active (gluco)mannan synthases share this VET motif with CSLCs and a number of other highly conserved motifs (Robert et al., 2021), suggesting that CSLAs and CSLCs could elongate polymers via similar mechanisms. While an initial catalytic domain swap of Arabidopsis CSLA2 with that of the nasturtium (*Tropaeolum majus*) CSLC4 was non-functional compared to the parental enzymes (Robert et al., 2021), structural data and/or detailed *in vitro* characterization are still needed to understand the biochemistry of CSLA/Cs.

The new oligosaccharide profiling of Arabidopsis mutants indicates that *CSLA2* and *CSLA9* are required for the synthesis of two different GGM motifs (Yu et al., 2022). Mannanase digestion released only trace amounts of carbohydrates from the *csla2 csla9* double mutant. This suggests that there may be a functional and tissue-dependent specialization within the *CSLA* family, but raises new questions about the roles of other CSLAs in Arabidopsis (Voiniciuc, 2022). The latest data show that *CSLA9*-dependent acetylated glucomannan motifs are found in PCW-rich tissues such as leaves (Yu et al., 2022), while *CSLA2* is required for repeating Glc-Man domains regularly substituted Gal side chains (discussed in next section). However, it is still unclear how GGM backbone patterns are defined. Based on synthetic biology approaches in yeast, MANNAN SYNTHESIS-RELATED (MSR) proteins from the GT106 family modulate the activities of CSLAs to enhance the elongation of (*gluco*)mannan (Robert et al., 2021, Voiniciuc et al., 2019). Even when co-expressed with MSR1 to produce glucomannan in yeast cell walls, Arabidopsis CSLA2 was unable to generate glucomannan with the high Glc to Man molar ratio found in seed mucilage (Voiniciuc et al., 2015b, Yu et al., 2018). Therefore, additional proteins likely function together *in vivo* to define GGM patterns.

## Substitution of mannan and XyG

**Fig. 1:**
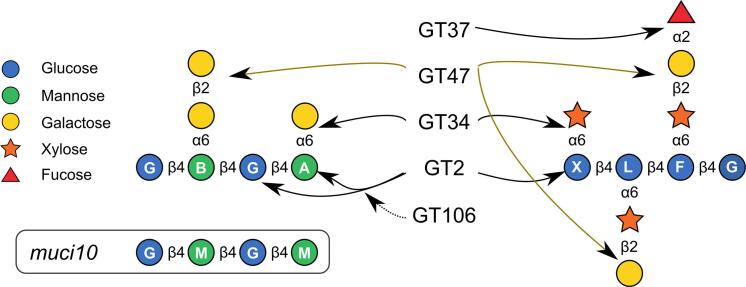
Schematic of GT families required for branched mannan and XyG biosynthesis. A highly branched motif found in Arabidopsis cell walls is shown for each type of polymer, and their biosynthesis requires related CAZy proteins. Models and single-letter nomenclature (Julian and Zabotina, 2022, Pauly and Keegstra, 2016, Yu et al., 2022) denotes the backbone unit and any associated branches, e.g. B for the disaccharide-decorated Man. Two different GT47 enzymes are required to add β-1,2-Gal to the different positions of XLFG. Characterized enzymes for each GT family are shown in Table 1.

The backbones of mannans and XyG can be substituted by GT34 enzymes with α-1,6-linked Gal or xylosyl (Xyl), respectively, or can be *O*-acetylated at the C-2 and C-3 positions (Pauly and Keegstra, 2016, Voiniciuc, 2022). Single α-1,6-Gal units are attached to Man by MANNAN α-GALACTOSYLTRANSFERASE1/MUCILAGE-RELATED 10 (MAGT1/MUCI10) using UDP-Gal (Voiniciuc et al., 2015b, Yu et al., 2018), while XyG Xyl TRANSFERASES (XXT) use UDP-Xyl as to add regular patterns *in vivo* (Culbertson et al., 2018). Interestingly, some XXTs can add additional Xyl residues on glucan oligosaccharides *in vitro* or even use other donor sugars such as UDP-Gal (Julian and Zabotina, 2022). This suggests that GT34 activity in plant cells is partly determined by donor substrate availability, and that the mannan-related enzyme specificities should be further explored. The Xyl side chains are usually further substituted with single β-1,2-Gal (abbreviated L) or β-1,2-Gal-α-1,2-Fucose (abbreviated F) units in Arabidopsis (Pauly and Keegstra, 2016). While XyG subunits as complex as XLFG are natively found in most Arabidopsis tissues (Fig. 1) and have been easy to identify based on characteristic mass spectra (Pauly and Keegstra, 2016), GGM oligosaccharides have been more challenging to fingerprint. Back in 2015, the significant reduction of terminal-Gal and 2-Gal linkages in mucilage extracted from *muci10* mutant seeds raised the hypothesis that β-1,2-Gal-α-1,6-Gal disaccharides may partly decorate glucomannan in Arabidopsis mucilage. Prompted by the previous evidence that β-galactosylation of α-Gal residues on XyG is catalyzed by GT47 enzymes such as MURUS3 (MUR3; *murus* means wall in Latin) and XYLOGLUCAN l-SIDE CHAIN GALACTOSYLTRANSFERASE POSITION2 (XLT2; reviewed by Pauly and Keegstra, 2016), Yu et al., (2022) elegantly showed that MANNAN β-GALACTOSYLTRANSFERASE1 (MBGT1), belonging to an uncharacterized clade of this CAZy family, adds β-1,2-Gal onto GGM (Fig. 1). While these β-Gal side chains were not abundant enough to be detected by solid state NMR, the native expression of β-galactosidases could be reducing the frequency of β-Gal observed in some cell walls (Yu et al., 2022).

## Unique and additive effects of mannan and XyG mutants

For the synthesis of GGM in the Arabidopsis seed mucilage, MAGT1/MUCI10 may function in a complex with CSLA2 to modulate its stability or activity because the glucomannan elongation is strongly reduced in *muci10* mutants. Similarly, the elongation of insoluble β-1,4-glucan by CSLC4 in yeast was boosted by XXT1 co-expression (Cocuron et al., 2007, Robert et al., 2021). The *csla2* and *muci10* increased the density of mucilage polysaccharides yet reduced the amount of crystalline cellulose relative to wild-type plants (Voiniciuc et al., 2015b). Biophysical changes associated with the loss of mannan branches have not been quantified, but we hypothesize the mutants such as *muci10* would enhance cell wall rigidity to mechanical stress, despite being more susceptible to enzymatic degradation by endo-mannanases and cellulases (Voiniciuc et al., 2015b). The effects of Indeed, GGM is more important for cellulose deposition than branched xylan (Voiniciuc et al., 2015a, Yang et al., 2020), another hemicellulose present in mucilage, while XyG-deficient *xxt1 xxt2 xxt5* triple mutant seeds have wild-type mucilage staining (Voiniciuc et al., 2015a). Even the loss of several microtubule-related proteins that guide cellulose synthase movement (Yang et al., 2022) caused less severe cellulose defects than GGM-deficient seeds. Therefore, mannans (akin to XyG in tip-growing cells; Kim et al., 2020) have unique roles in some tissues (Voiniciuc, 2022).

Although XyG is the most abundant hemicellulose in the PCW of eudicots, the lack of detectable XyG in quintuple *cslc* mutants (Kim et al., 2020) and triple *xxt* mutants (Zabotina et al., 2012) only results in minor growth defects. The hypothesis that other polysaccharides can compensate for the loss of XyG in these plants has lacked evidence until recently. While XyG-deficient mutants do not have clearly altered expression of *CSLA* genes (Kim et al., 2020, Sowinski et al., 2022), *xxt1 xxt2* double mutants have now been reported to have increased glucomannan and pectin content (Sowinski et al., 2022), potentially due to reduced polysaccharide turnover. Changes in glucomannan content were not detected by an independent team using oligosaccharide profiling (Yu et al., 2022), but a third group found significant changes in the mass distribution profiles of mannans labelled by the LM21 antibody in XyG-deficient mutants (Sathitnaitham et al., 2021). The higher order mutants generated by Yu et al. (2022) provide the strongest evidence that β-galactosylated GGM and XyG have functionally overlapping roles in some PCWs. The *csla2 xxt1 xxt2* triple mutant reduced cell elongation and impaired cellulose organization, resulting in shorter plants and siliques compared to the respective mutants backgrounds (Yu et al., 2022). Furthermore, *mbgt1 mur3-3* double mutant was exacerbated the cabbage-like phenotype of the *mur3-3* single mutant, suggesting that β-galactosylated GGM partially compensated for the XyG deficiency during early growth (Yu et al., 2022). Further comparative studies of branched mannans and XyG are now needed to address several puzzles that remain at the cell surface. Exploring the biodiversity of proteins and their complexes that add or modify these patterned hemicelluloses will reveal new insights into their secretion and interaction with cellulose microfibrils.

## CRediT authorship contribution statement

**Annika Grieß-Osowski:** Writing – original draft, Writing – review & editing. **Cătălin Voiniciuc:** Writing – review & editing, Visualization.

## Declaration of Competing Interest

The authors declare that they have no known competing financial interests or personal relationships that could have appeared to influence the work reported in this paper.
